# LAG3 facilitates MHC Ⅱ trogocytosis with assistance of the ER-PM junction

**DOI:** 10.7555/JBR.39.20250144

**Published:** 2025-05-27

**Authors:** Zibin Wang, Jing Wang, Wene Zhao, Wen Liu

**Affiliations:** 1 Analysis and Test Center, Nanjing Medical University, Nanjing 211166, China.; 2 Department of Reproductive Medicine, Zhongda Hospital, School of Medicine, Southeast University, Nanjing 210009, China; 3 School of Life Sciences, Nanjing University, Nanjing 210023, China

Dear Editor,

Lymphocyte activation gene 3 (LAG3), the third established target for immune checkpoint blockade therapy, suppresses T cell function by binding to major histocompatibility complex class Ⅱ (MHC Ⅱ). Despite its significant therapeutic potential in cancer immunotherapy and the substantial attention it has received from academia and industry, the molecular mechanisms of LAG3-mediated immunosuppression remain poorly understood, primarily because of its unique ligand-binding characteristics and intracellular domains ^[ 1] ^. Recent studies have advanced our understanding of LAG3 function. Maruhashi *et al* ^[ 2]^ have demonstrated that stable peptide-MHC Ⅱ, rather than fibrinogen-like protein (FGL1), serves as the functional ligand for LAG3-mediated T cell suppression in both autoimmunity and anti-cancer immunity. Jiang *et al* ^[ 3]^ have revealed the molecular mechanism by which LAG3 is activated by MHC Ⅱ-mediated ligand engagement, with ubiquitination activating LAG3 by releasing its cytoplasmic tail (CT) from the membrane. Notably, LAG3 associates with the T cell receptor (TCR)-CD3 complex and traffics to the immunological synapse (IS), where clustering of its CT through a phase separation mechanism disrupts interactions between coreceptors CD4/CD8 and the kinase Lck, thereby impairing TCR signaling and reducing T cell activation ^[ 4] ^. These studies have deepened our understanding of LAG3's immunomodulatory mechanisms and highlighted the need to elucidate the molecular mechanisms underlying the biological function of the LAG3-MHC Ⅱ interaction in a true cell-cell contact system.

Using reconstituted intercellular conjugation assays and high-resolution imaging, we investigated LAG3's effects on cellular ultrastructure. When fused to enhanced green fluorescent protein (LAG3-EGFP) and expressed in HEK293T cells (LAG3 ^+^ 293T), LAG3 accumulated at the intercellular contact interface, indicating a preference for homotypic trans interactions ( ***Supplementary Fig. 1A***). To validate this, conjugates were established using HEK293T cells expressing different fluorescent LAG3 variants, demonstrating a notable co-localization of green and red fluorescence at cell junctions ( ***Fig. 1A***). Structural studies of human and murine LAG3 ectodomains, or of LAG3 complexed with MHC Ⅱ, have revealed dimeric assemblies mediated by domain 2 (D2) ^[ 5– 7] ^. Our results demonstrate a potential homotypic trans interaction of LAG3 between cells, indicating possible immunomodulatory roles beyond classical ligand engagement.

**Figure 1:**
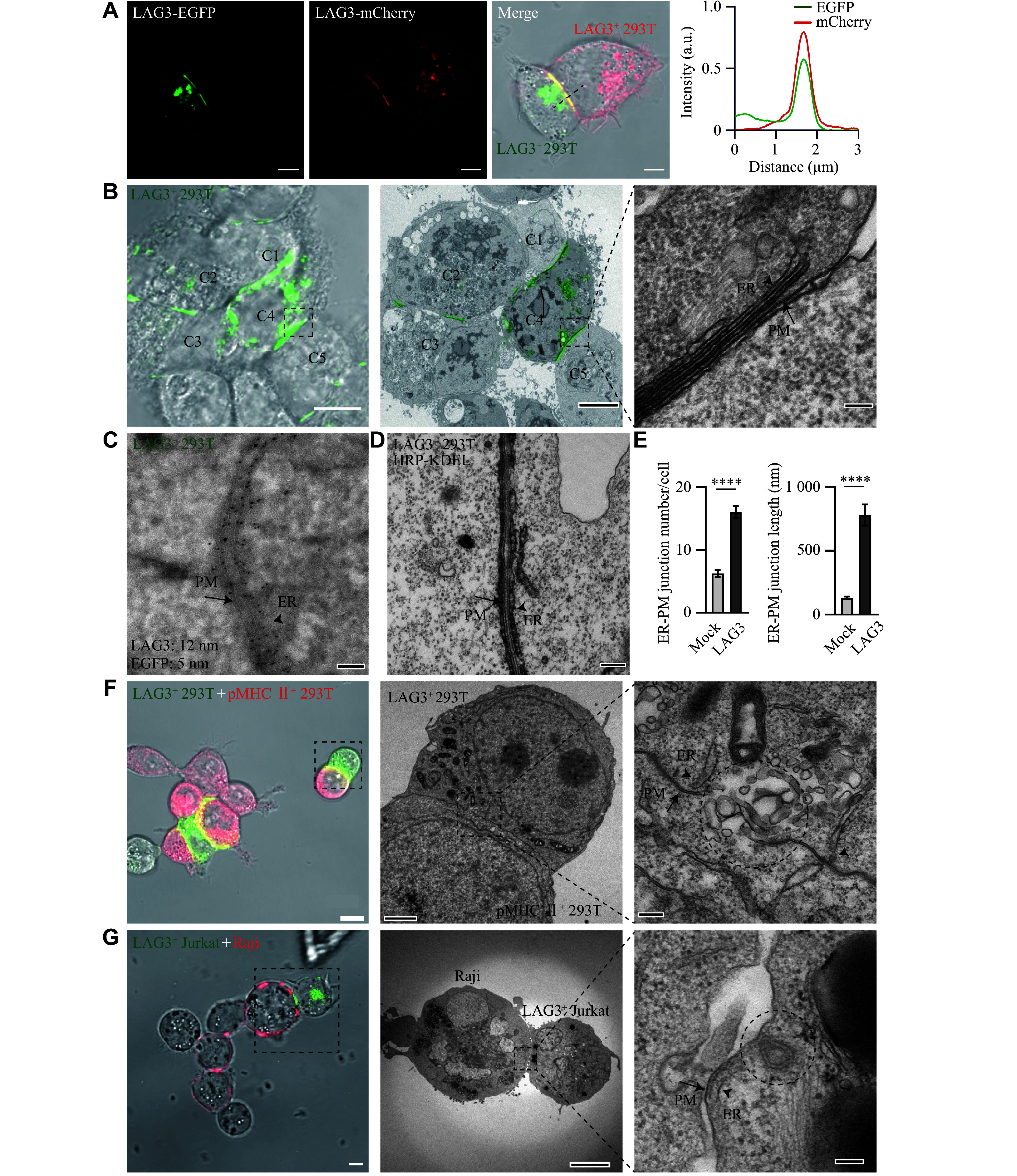
LAG3 facilitates MHC Ⅱ trogocytosis with assistance of the endoplasmic reticulum (ER)-plasma membrane (PM) junction. A: Representative confocal images of conjugates of LAG3-EGFP-expressing HEK293T cells with LAG3-mCherry-expressing HEK293T cells. Fluorescence intensities of EGFP and mCherry along the dashed line in the merged images are shown. Scale bar, 2 μm. B: Representative correlative light and electron microscopy (CLEM) images (left, fluorescence image; middle, transmission electron microscopy image; right, magnified TEM image of the region of interest outlined by the dashed box) of LAG3-EGFP-expressing HEK293T cells. Cells C1–C5 are indicated. Scale bars: 10 μm (left/middle), 100 nm (right). C: Representative immuno-electron microscopy images of LAG3-EGFP-expressing HEK293T cells. LAG3 and EGFP proteins were labeled with 12 nm and 5 nm colloidal gold antibodies, respectively. Scale bar, 100 nm. D: Representative TEM images of LAG3-EGFP and HRP-KDEL co-expressed HEK293T cells. Scale bar, 100 nm. E: Quantification of ER-PM junction number ( *n* = 20) and length ( *n* = 50) in TEM images of mock or LAG3-EGFP and HRP-KDEL co-expressed HEK293T cells. Data are presented as mean ± standard error of the mean. ^****^ *P* < 0.0001 by unpaired Student's *t*-test. F and G: Representative CLEM images (left, fluorescence image; middle, transmission electron microscopy image; right, magnified TEM image of the region of interest outlined by the dashed box) of conjugates of LAG3 ^+^ 293T cells with pMHC Ⅱ ^+^ 293T cells (F) and LAG3 ^+^ Jurkat cells with Raji cells (G). The trogocytosis sites are outlined by dashed circles. Scale bars: 5 μm (left; F and G), 200 nm (middle; F), 500 nm (middle; G), 100 nm (right; F and G). The ER (arrowhead) and PM (arrow) form membrane contact sites (MCSs; A–D, F, and G). Abbreviations: LAG3, lymphocyte activation gene 3; EGFP, enhanced green fluorescent protein.

Transmission electron microscopy (TEM) revealed an intriguing ultrastructure: endoplasmic reticulum (ER)-like tubular membranes juxtaposed closely with plasma membranes (PM) at intercellular contacts in LAG3 ^+^ 293T cells ( ***Supplementary Fig. 1B***). Since conventional TEM imaging is unable to simultaneously acquire fluorescence signals and determine cellular ultrastructure at the localization of target proteins, we employed correlative light and electron microscopy (CLEM) ^[ 8]^ for further study. The CLEM imaging of LAG3 ^+^ 293T cells revealed the formation of membrane contact sites (MCSs) of ER-like tubular membrane with the PM at the LAG3 accumulation sites ( ***Fig. 1B***), forming the ER-PM junction, which is important for inter-organelle communication ^[ 9] ^. Immuno-electron microscopy (immuno-EM) with double labeling for LAG3 and EGFP demonstrated colocalization of the LAG3 CT with the ER-like membrane and the LAG3 ectodomain with the PM ( ***Fig. 1C***). Additionally, ER identification was achieved through the ER marker SEC61β ( ***Supplementary Fig. 1C***) and a horseradish peroxidase (HRP)-tagged ER retention motif (HRP-KDEL), which exhibited high electron density in TEM images ( ***Fig. 1D***). Quantitative analysis showed that LAG3 ^+^ 293T cells had significantly more and longer ER-PM junctions than controls ( ***Fig. 1E***; ***Supplementary Fig. 1D*** and ***1E***). Importantly, similar ER-PM junctions were observed between conjugates of LAG3-expressing Jurkat cells (LAG3 ^+^ Jurkat) and MHC Ⅱ-expressing Raji cells ( ***Supplementary Fig. 1F***), suggesting that the ER-PM junctions induced by LAG3 may occur at the ISs.

To elucidate the mechanism of LAG3-mediated ER-PM junction formation, we determined the oligomerization of LAG3 using bimolecular fluorescence complementation (BiFC). BiFC analysis revealed that oligomerized LAG3 localized to the cell surface and colocalized with the ER ( ***Supplementary Fig. 2A***). TEM further validated ER-PM junction formation ( ***Supplementary Fig. 2B***). Domain truncation studies (LAG3ΔCT and LAG3ΔD1D2) demonstrated that the CT domain was essential for LAG3 trafficking to the cell surface and ER-PM junction formation, as LAG3ΔCT remained cytoplasmic and failed to induce junctions ( ***Supplementary Fig. 2C*** and ***2D***). The D1D2 domains, while required for homo-trans interaction and ligand engagement, were dispensable for junction formation, as LAG3ΔD1D2 retained surface localization and enhanced ER-PM junctions ( ***Supplementary Fig. 2E*** and ***2F***). Additional control experiments demonstrated that the oligomerization of CD4 was insufficient to induce ER-PM junctions ( ***Supplementary Fig. 2G*** and ***2H***), excluding fluorescent protein artifacts. Together, these findings demonstrate that LAG3 oligomerization and its CT domain are necessary and sufficient for ER-PM junction formation, independent of ligand engagement.

Recent work by Wakamatsu *et al* ^[ 10]^ showed that LAG3-mediated trogocytosis reduced MHC Ⅱ expression on antigen-presenting cells, impairing CD4 ^+^ T cell activation. Trogocytosis, a conserved intercellular material exchange process occurring at the IS, is characterized by the transfer of surface molecules and membrane fragments between immune cells ^[ 11] ^. In the current study, CLEM imaging captured the internalization of MHC Ⅱ ^+^ membrane fragments by LAG3-expressing cells ( ***Fig. 1F*** and ***1G***), suggesting a trogocytosis-like internalization of membrane fragments. Notably, ER-PM junctions formed near trogocytosis sites ( ***Fig. 1F*** and ***1G***), implying their potential role in this process. While cytotoxic T lymphocyte-associated protein 4 (CTLA4) and programmed cell death protein 1 (PD1) were also reported to mediate trogocytosis ^[ 12– 13] ^, the current study provides direct ultrastructural evidence of LAG3's involvement. Though the mechanisms of trogocytosis remain unclear, the ER-PM junction may facilitate it by promoting endocytosis ^[ 14– 15] ^. As critical hubs for inter-organelle communication, MCSs are areas of close apposition between the membranes of two organelles and are important for physiological functions in mammalian cells. In particular, the ER-PM junction is specific for Ca ^2+^-release-activated Ca ^2+^ (CRAC) channels, which generate sustained Ca ^2+^ signals that are essential for antigen-stimulated T lymphocyte activation and proliferation ^[ 9, 16– 17] ^. Thus, we speculate that LAG3 may modulate Ca ^2+^ signaling at the IS by mediating the formation of the ER-PM junction, thereby exerting immunomodulatory functions and promoting trogocytosis. While the unstructured CT of LAG3 belongs to a class of disordered proteins that are potentially involved in the formation of MCSs ^[ 18] ^, the exact mechanism of LAG3-mediated ER-PM junctions in trogocytosis requires further investigation.

In conclusion, our multimodal high-resolution imaging and ultrastructural analysis revealed a previously unrecognized biological function of LAG3 in inducing ER-PM junction formation, providing novel insights into its immunoregulatory function.

Yours sincerely,

Zibin Wang ^1,✉^, Jing Wang ^2^, Wene Zhao ^1^, and Wen Liu ^3^

^1^Analysis and Test Center, Nanjing Medical University,

Nanjing, Jiangsu 211166,

China;

^2^Department of Reproductive Medicine, Zhongda Hospital, School of Medicine, Southeast University,

Nanjing, Jiangsu 210009,

China;

^3^School of Life Sciences, Nanjing University,

Nanjing, Jiangsu 210023,

China.

^✉^Corresponding author: Zibin Wang. E-mail: wangzibin@njmu.edu.cn.

## Additional information

The online version contains supplementary materials available at http://www.jbr-pub.org.cn/article/doi/10.7555/JBR.39.20250144.
