# Supplementary material for: LAG3 facilitates MHC Ⅱ trogocytosis with assistance of the ER-PM junction
Source: J Biomed Res. 2025 May 27;40(1):89–92. doi: 10.7555/JBR.39.20250144 (PMC12794176; doi:10.7555/JBR.39.20250144)
Supplement: Supplementary file 1 — The online version contains supplementary materials available at http://www.jbr-pub.org.cn/article/doi/10.7555/JBR.39.20250144. [file jbr-40-1-89-S1.pdf]

Available online at www.jbr-pub.org.cn

Open Access at PubMed Central

Journal of Biomedical Research, 2026 40(1): 1–2

Supplementary Data

JBR

LAG3 facilitates MHCII trogocytosis with assistance of the ER-PM junction

Zibin Wang[1,✉], Jing Wang[2], Wene Zhao[1], Wen Liu[3]

[1]*Analysis and Test Center, Nanjing Medical University, Nanjing, Jiangsu 211166, China;*
[2]*Department of Reproductive Medicine, Zhongda Hospital, School of Medicine, Southeast University, Nanjing, Jiangsu 210009, China;*
[3]*School of Life Sciences, Nanjing University, Nanjing, Jiangsu 210023, China.*

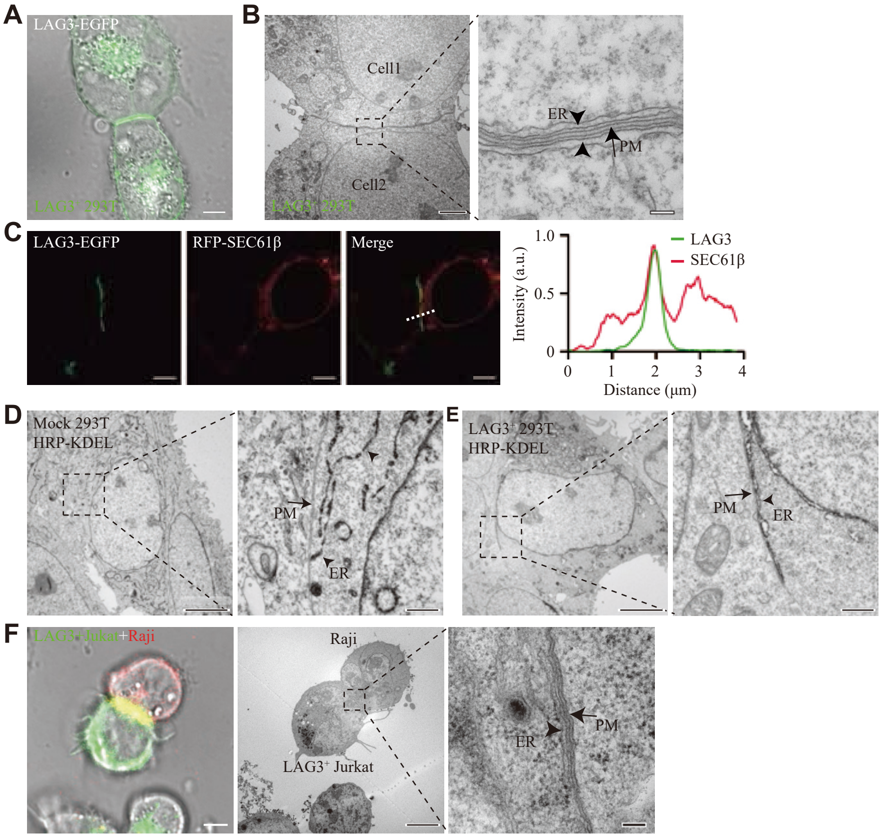

Supplementary Fig. 1 **LAG3 induced the formation of ER-PM junctions.** A: Representative confocal images of LAG3-EGFP-expressing HEK293T cells. Scale bar, 5 μm. B: Representative transmission electron microscopy (TEM) images of LAG3-EGFP-expressing HEK293T cells. The ER (arrowhead) and PM (arrow) form MCSs. Scale bar, 2 μm (left panel), 100 nm (right panel). C: Representative confocal images of LAG3-EGFP and RFP-SEC61β co-expressed HEK293T cells. Fluorescence intensities of EGFP and RFP are shown along the dashed line in the merged image. Scale bar, 5 μm. D and E: Representative TEM images of mock (D) and LAG3-EGFP and HRP-KDEL co-expressed HEK293T cells (E). The ER (arrowhead) and PM (arrow) form MCSs. Scale bar, 2 μm (left panel), 500 nm (right panel). F: Representative CLEM images of conjugates of LAG3+ Jurkat with Raji cells. Left panel, fluorescence image; middle panel, TEM image; right panel, magnified TEM image of the region of interest outlined by a dashed box. The ER (arrowhead) and PM (arrow) form MCSs. Scale bar, 5 μm (left and middle panels), 100 nm (right panel).

✉Corresponding author: Zibin Wang. E-mail: wangzibin@njmu.edu.cn.

Received: 01 April 2025; Revised: 02 May 2025; Accepted: 08 May 2025; Published online: 27 May 2025

CLC number: R392.1, Document code: B

The authors reported no conflict of interests.

This is an open access article under the Creative Commons Attribution (CC BY 4.0) license, which permits others to distribute, remix, adapt and build upon this work, for commercial use, provided the original work is properly cited.

© 2026 by Journal of Biomedical Research.

https://doi.org/10.7555/JBR.39.20250144

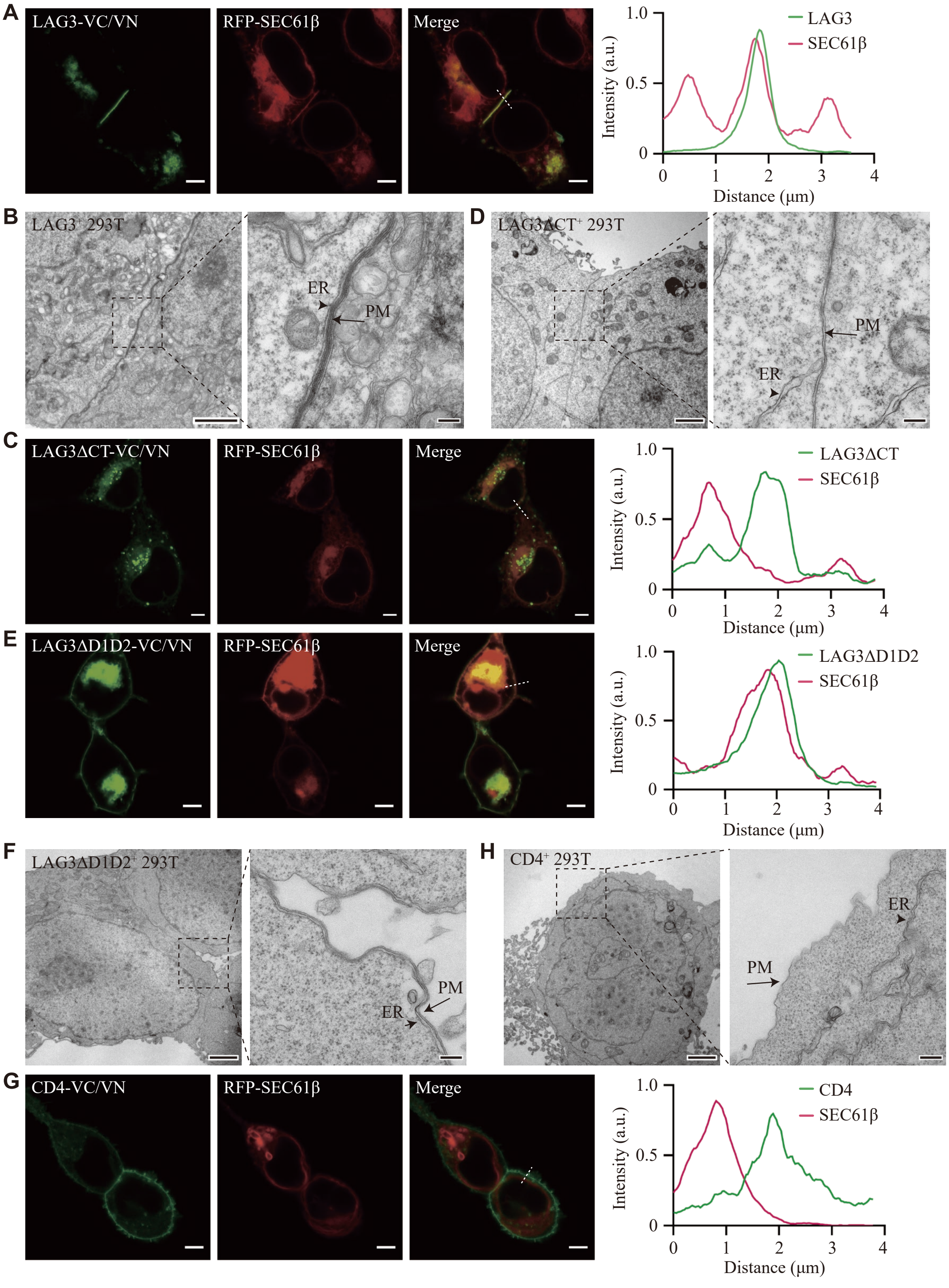

Supplementary Fig. 2 **Oligomerized LAG3 on the cell surface induces the formation of ER-PM junctions.** A, C, E, and G: Representative bimolecular fluorescence complementation (BiFC) images of full-length LAG3 (A), truncated CT (C), truncated D1D2 domains (E), and CD4 (G). The yellow fluorescent protein Venus was cleaved into two parts: C-terminal (VC) and N-terminal (VN), which were fused to the C-terminus of LAG3, truncated LAG3, and CD4, respectively. They were then co-expressed with the ER marker SEC61β in HEK293T cells. Fluorescence intensities of Venus and RFP are shown along the dashed line in the merged images. Scale bar, 5 μm. B, D, F, and H: Representative transmission electron microscopy (TEM) images of full-length LAG3 (B), truncated CT (D), truncated D1D2 domains (F), and CD4 (H) in HEK293T cells. The ER (arrowhead) and PM (arrow) form MCSs. Scale bar, 2 μm (left panels), 200 nm (right panels).
